# Hemp seed (*Cannabis sativa* L.) enriched pasta: Physicochemical properties and quality evaluation

**DOI:** 10.1371/journal.pone.0248790

**Published:** 2021-03-18

**Authors:** Dorota Teterycz, Aldona Sobota, Dominika Przygodzka, Paulina Łysakowska

**Affiliations:** 1 Department of Plant Food Technology and Gastronomy, Division of Engineering and Cereals Technology, University of Life Sciences in Lublin, Lublin, Poland; 2 Chair and Department of Forensic Medicine, Medical University of Lublin, Lublin, Poland; Higher Institute of Applied Sciences and Technology of Gabes University of Gabes, TUNISIA

## Abstract

Hemp seed (*Cannabis sativa* L.) contain large amounts of nutrients, e.g. protein, dietary fiber, minerals, and unsaturated fatty acids, which make them a good fortifying component in food production. The aim of the present study was to determine the effect of hemp addition on the physicochemical properties, cooking quality, texture parameters and sensory properties of durum wheat pasta. The samples were fortified with 5–40% of commercially available hemp flour or 2.5–10% of hemp cake obtained from hemp seed oil pressing. Our study showed that the addition of hemp seed raw materials led to an increase in the protein, total dietary fiber (TDF), ash and fat content in the pasta samples. Due to its lower granulation and higher nutritional value, hemp flour was found to be a better raw material for the fortification of pasta than hemp cake. Pasta enriched with hemp flour at the level of 30–40% contains 19.53–28.87% d.m. of protein and 17.02–21.49% d.m. of TDF and according to the EU, a definition can be described as a high-protein and high-fiber products. All enriched pasta samples were also characterized by safe Δ-9-tetrahydrocannabinol (THC) and cannabidiol (CBD) content, and their sensory properties were accepted by consumers.

## 1. Introduction

In recent years, increasing interest in vegan and vegetarian diets has been observed. A problem in this type of diets is to provide the appropriate amount of protein. Consumers are looking for new plant-based raw materials that could be a good source of protein. One of the high-protein raw materials, which is increasingly becoming part of a healthy balanced human diet is hemp (*Cannabis sativa* L.) [1]. Interest in this plant has been growing since 1996 when varieties with THC (Δ-9-tetrahydrocannabinol) content below 0.3% were approved for cultivation in EU countries. Currently, this limit is reduced to 0.2% [2].

Hemp seeds are a rich source of nutrients. They contain about 35% oil, 25% of protein, 28% of total dietary fiber (TDF) an 5.6% of minerals [3]. Hemp protein consists mainly of a storage protein edestin (11S globulin), which accounts for 60–80% of the total protein in this raw material. Albumin (2S) constitutes the rest of the protein fraction [4]. Hemp seed protein is highly digestible, ranging from 84–86% for whole seeds to 83–92% for seed meal, but at the same time has a fairly low Protein Digestibility-Corrected Amino Acid Score (PDCASS), 49–54% for whole hemp seed and 46–51% for hemp seed meal. This parameter is limited by relatively low content of essential amino acids such as lysine and tryptophan [5]. However, studies indicate that hemp protein digestion may produce bioactive peptides with antioxidant, antihypertensive, antimicrobial and cytomodulatory activity [4]. The hemp seed is a rich source of TDF. The dominant fraction of TDF (about 80%) is insoluble dietary fiber (IDF). The main minerals found in hemp seeds are phosphorus (1160 mg/100 g), potassium (859 mg/100 g), magnesium (483 mg/100 g) and calcium (145 mg/100 g) [3]. The main use of hemp seed processing is oil production [6]. Oil from hemp seeds can be a very valuable component of the diet due to its high content of unsaturated fatty acids and an appropriate omega-6 to omega-3 ratio (3:1) [7]. A by-product of hemp oil production, i.e. hemp oil cake, due to high content of protein (33.5%), dietary fiber (42.6%) and minerals (7.2%) can also be used to improve the nutritional value of foods [8, 9]. Hemp cake is usually grounded, which makes it a highly versatile raw material used, among others, in the cereal industry [9, 10].

Hemp-based food must meet the THC content requirements. According to Fresh Hemp Foods Ltd. [11], the daily THC total intake should not exceed 1–7 μg/kg body weight, depending on the country’s restriction. It is important not to exceed the recommended dose, as it has been proven that regular intake of THC is associated with serious side effects, including cognitive deficiency, anxiety, paranoia, chronic psychosis, and addiction [12]. However, research shows that Δ-*9*-*tetrahydrocannabinol* accounts for only 10% of the total THC content in the products, whereas the other part is Δ-*9-tetrahydrocannabinol carboxylic acid* (THCA), which has no psychoactive effect. The conversion of THCA to THC only takes place during long-term heat treatment at 115°C [13]. These conditions cannot be obtained when drying or cooking pasta, so there is most likely no danger of THCA becoming psychoactive form, THC in such product. Another important component of cannabis seeds for which intake limits are applied is cannabidiol (CBD). It has no psychoactive effect, but it is recommended that the CBD content should not exceed 7.5 mg/100 g of the food product [14] or less consumption than 20 mg per day for an adult [15]. CBD has a neuroprotective effect; hence, it can be used in neurodegenerative diseases or epilepsy. In natural medicine, it is also used as an analgesic and anti-inflammatory medicine [16].

In recent years, research has been undertaken to assess the possibility of using hemp raw materials in products such as bread [10, 17] and cookies [18]. However, there is no research on the possibility of using hemp component for the fortification of the nutritional value of durum wheat pasta. The aim of the present study was to evaluate the effect of the hemp flour or hemp cake addition on the physicochemical properties, texture parameters, cooking and sensory qualities of the pasta.

## 2. Materials and methods

### 2.1. Raw materials

The raw materials used in the study included durum semolina produced by Julia Malom (Kunszallas, Hungary), commercially available defatted hemp flour (BioPlanet, Leszno, Poland), and hemp cake prepared from seed (cultivar Finola) by Galeria Konopi (Zabrze, Poland) and obtained after cold press extraction of hemp oil. The cold pressing process was carried out using a screw press (Press M222/15F, Miramar, Nowa Wieś, Poland), with screw speed 30 rpm and barrel temperature 45°C.

### 2.2. Fractional composition of raw materials

The particle size of the semolina durum and hemp raw materials were determined using the method described by Sobota et al. [19]. The degree of fragmentation of the raw materials was estimated by means of sieve analysis. A sample of 100 g was sieved for 10 min with a sieve shaker (ZBPP, Bydgoszcz, Poland) using sieves of 410, 315, 250, 160, 125, 80 μm. The equivalent diameter was determined according to the formula given by Kasprzak et al. [20].

### 2.3. Production of pasta

Pasta samples were produced in laboratory semi-technical scale conditions using a pasta extruder MAC 30S-Lab (ItalPast, Fidenza, Italy). The level of addition of hemp flour and hemp cake was established by means of preliminary tests determining a maximum addition that did not interfere with the technological process (Table 1). The raw materials, i.e. durum semolina, hemp flour, hemp cake, and water, were combined in appropriate proportions to obtain 3 kg of each sample. The amount of water addition ensured 32% moisture content in the mixture. The addition of water to the samples with hemp raw materials was higher due to their lower moisture content. The raw materials were mixed together for 15 minutes in a premixer. Next, pasta dough was mixed and extruded in vacuum conditions at low temperature (28°C). The extrusion pressure was 110 bar. A Teflon die was used for pasta production. Samples of pasta (Fusilli) were dried in an EAC 30-Lab pasta dryer (ItalPast, Fidenza, Italy) using a low-temperature profile (35–55°C) and 85–55% relative humidity of the drying air.

**Table 1:** Model of experiment.

Pasta sample	Semolina	Hemp flour	Hemp cake	Water addition
	%			g/kg of raw mixture
**CON**	**100**	0		306
**HF-5**	95	5		309
**HF-10**	90	10		313
**HF-15**	85	15		316
**HF-20**	80	20		320
**HF-25**	75	25		323
**HF-30**	70	30		327
**HF-35**	65	35		330
**HF-40**	60	40		333
**HC-2.5**	97.5		2.5	307
**HC-5**	95		5	309
**HC-7.5**	92.5		7.5	311
**HC-10**	90		10	312

CON–control sample. HF–hemp flour. HC–hemp cake.

### 2.4. Chemical analysis of raw materials and pasta samples

The chemical composition was analyzed to determine the content of protein, fat, dietary fiber, moisture and ash. The total dietary fiber (TDF) content in the pasta and raw material samples was determined with the enzymatic methods of the American Association of Cereal Chemists (AACC) and the Association of Official Agricultural Chemists (AACC, 2000: Methods 32–05 and 32–21, AOAC, 991.43, AOAC 985–29). The water content was determined with the air-oven method (AACC 44-15A). The ash content was determined with the basic method (AACC 08–01) [21, 22].

The protein content was determined using a Kjeltec TM8400 machine with the ASN (Application Sub Notes) 3100 application. The total fat content was determined after acid hydrolysis by continuous extraction. The SoxtecTM8000 with AN 310 and hexane as a solvent was used. The available carbohydrate content was calculated from the difference: 100 − (weight in grams [protein + fat + TDF + ash]) in 100 g of dry matter of the pasta or raw material. The content of wet gluten in the wheat raw material was determined with the handwashing method (AACC 38–10) (AACC, 2000). The energy value for the pasta samples was calculated using the modified Atwater coefficient (protein—4 kcal, carbohydrates—4 kcal, fat—9 kcal, TDF—2 kcal). The determination of the chemical composition of the raw materials and pasta was conducted in three replications.

The THC and CBD content in the raw materials was determined by means of a high-performance liquid chromatograph (HPLC 1260 Agilent Technologies, Germany), a Poroshell 120 EC-C18, 3.0 × 50 mm; 2.7 μm column (Agilent Technologies, USA), and a mass spectrometer (triple quadrupole type (QqQ 6460, Agilent Technologies, USA) with APCI negative/positive ionisation.

### 2.5. Cooking quality of pasta samples

Cooking quality parameters such as minimal cooking time, weight increase index, and cooking losses were determined according to the methodology proposed by Teterycz et al. [23].

### 2.6. Water solubility index and water absorption index

The water solubility index (WSI) and the water absorption index (WAI) were determined according to the method described by Zarzycki et al. [24].

### 2.7. Texture characteristics assessment

The pasta samples were cooked according to the minimum cooking time set. The test material prepared in this way was subjected to instrumental testing using a Zwick/Roell testing machine, cooperating with a computer. A double compression test TPA (Texture Profile Analysis) was performed. The specimens were compressed at the speed of the device head movement of 50 mm-min^−1^ up to 50% of the height of the sample. The determination for each of the samples was performed in 5 repetitions. The basic texture parameters (hardness, springiness, cohesiveness, chewiness) were calculated on the basis of the measurement curve.

### 2.8. Organoleptic analysis

The organoleptic properties of cooked pasta were assessed by an 18-person (25–50 years old). The panelists were selected from habitual consumers of pasta based on the following criteria: willingness to participate (voluntary written declaration of participation in the research), good health, nonsmoker, and not allergic to gluten/wheat products. Before the test, the participants were trained and tested for determination of their sensory sensitivity. Pasta samples were cooked in distilled water for the prescribed minimal cooking time and presented on white plates. The consumers were provided with plain water (at room temperature) to clean their palate before the test and between the pasta samples. The sensory evaluation was conducted in a laboratory at led lighting and ambient temperature.

The organoleptic characteristics, i.e. appearance, color, taste, smell, springiness, hardness and adhesiveness were evaluated on a scale of 1 to 5, where 5 was the maximum value of the parameters. An average note was calculated from all the assessments.

### 2.9. Ethics

The Bioethics Commission (Poland) decided that the study did not require the consent of the Commission. The sensory evaluation of the pasta samples was carried out after determination of the content of cannabinoids and after confirmation of their safe level by preliminary in vivo tests conducted by two persons. Only adult volunteers who declared that they were healthy, non-smokers, and not allergic to gluten, were allowed to take part in the study. The panelist agreed in writing to participate in the study. All participants were informed about every detail, including the characteristics and safety of the product. The study did not involve any risk of pain or discomfort for its participants. The test was designed to minimize the amount of consumed products and the panelist could leave the research at any time. The participants evaluated the products objectively and agreed to anonymous publication of their evaluation results.

### 2.9. Statistical analysis

The mean values and standard deviations were calculated. The results were statistically analyzed using one-way analysis of variance with replication (ANOVA, STATISTICA 13, StatSoft, Inc., Tulsa, USA). The significance difference between mean values was determined with the Tukey HSD test (p<0.05).

## 3. Result and discussion

Hemp cake used in the study was obtained by pressing the oil from seeds while the hemp flour was obtained from dehulled, grounded and solvent extracted defatted hemp seeds. The sieve analysis of the raw materials used to produce the pasta indicates a very high equivalent diameter of hemp cake (Table 2). This prevented the addition of this raw material at a level higher than 10% to the pasta, as the increased share of hemp cake would have plugged the dies. Hemp flour is a better raw material for pasta production not only because of the acceptable granulation but also because of the higher nutritional properties. This may be related to the different methods of defatting the seeds and thus the different fat content in both raw materials (7.79% d.m. in the hemp flour *vs*. 12.57% d.m. in the hemp cake; p < 0.05). Hemp flour was a better source of protein than hemp cake (35.47% d.m. *vs*. 34.19% d.m.; p < 0.05) (Table 2). Statistically significant differences in favour of the hemp flour were also noted for TDF, IDF, and ash content. In the hemp flour TDF, content was 46.44% d.m. while in the hemp cake 43.82% d.m. Since the bioactive substances THC and CBD found in hemp seed are highly lipophilic [25], the content of these substances was several times higher in the hemp cake than in the hemp flour. However, the THC content in both raw materials did not exceed the limits set by the European Union (0.2%).

**Table 2:** Physicochemical properties of raw materials.

Raw material	Equivalent diameter	Moisture	Ash	Protein	Fat	IDF	SDF	TDF	Carbo -hydrates*	THC	CBD
	μm	%	% d.m.							μg/100 g w.m.	
**Semolina**	268.28^c^±1.36	11.21^a^±0.12	0.81^c^±0.01	14.60^c^±0.02	1.27^c^±0.05	2.74^c^±0.04	1.50^b^±0.13	4.24^c^±0.09	79.08^a^±0.16	nd	nd
**Hemp flour**	303.10^a^±0.78	6.50^b^±0.01	7.79^a^±0.03	35.47^a^±0.29	7.79^b^±0.14	41.74^a^±0.12	4.70^a^±0.55	46.44^a^±0.44	2.60^b^±0.32	720.00^b^±15.00	1070.00^b^±22.00
**Hemp cake**	473.49^b^±1.52	6.79^b^±0.01	6.12^b^±0.01	34.19^b^±0.19	12.57^a^±0.25	39.98^b^±0.49	4.29^a^±0.55	43.82^b^±0.35	3.30^b^±0.29	5640.00^a^±16.00	2260.00^a^±25.00

*carbohydrate content calculated by difference. nd–not detected. IDF–insoluble dietary fiber. SDF–soluble dietary fiber. TDF–total dietary fiber. THC - Δ-9-tetrahydrocannabinol. CBD–cannabidiol. Means (n = 3) with different letters in the same column are significantly different (P < 0.05).

The nutrient content of pasta samples is given in Table 3. The content of individual nutrients varied statistically depending on the raw material composition of the sample. Each increase in the addition of the hemp flour resulted in an increase in the protein, fiber, fat, and ash content, compared to the control sample made from durum semolina (CON). The addition of the hemp cake also increased the content of the individual nutrients mentioned above. Similar results were noted in investigations of pasta enriched with flaxseed and coconut by-products (cake and flour). Pasta fortified with the flaxseed component up to 23% [26] or coconut residue up to 25% [27] had an improved nutritional composition and was characterized by a significantly higher content of protein, fat and ash, compared to the control sample.

**Table 3:** Chemical composition of pasta samples.

Pasta sample	Moisture	Ash	Protein	Fat	IDF	SDF	TDF	Carbo-hydrates*	Energy
	%	% d.m.							kcal/100 g d.m.
**CON**	10.16^abc^±0.08	0.79^j^±0.01	14.58^j^±0.12	1.25^i^±0.04	2.92^j^±0.09	1.51^e^±0.05	4.43^l^±0.14	78.95^a^±0.20	397.39^abc^±0.04
**HF-5**	10.15^abc^±0.13	1.21^h^±0.04	15.49^hi^±0.36	1.64^h^±0.13	4.62^hi^±0.18	1.74^de^±0.08	6.36^ji^±0.26	75.30^b^±0.40	395.48^c^±0.17
**HF-10**	10.69^ab^±0.27	1.52^f^±0.06	16.32^g^±0.27	2.00^g^±0.01	6.74^g^±0.14	1.76^de^±0.14	8.50^g^±0.28	71.66^c^±0.94	393.00^d^±0.54
**HF-15**	10.13^abc^±0.26	1.91^e^±0.08	17.20^f^±0.10	2.33^f^±0.16	8.55^f^±0.22	2.03^cd^±0.01	10.88^f^±0.23	67.68^d^±0.03	389.89^d^±0.45
**HF-20**	10.04^bc^±0.23	2.11^e^±0.09	17.90^e^±0.08	2.63^e^±0.10	10.44^e^±0.17	2.20^c^±0.05	12.64^e^±0.22	64.72^e^±0.35	387.87^e^±0.60
**HF-25**	10.45^abc^±0.03	2.59^d^±0.05	18.72^d^±0.10	2.91^d^±0.24	12.74^d^±0.16	2.28^bc^±0.08	15.02^d^±0.24	60.76^f^±0.15	384.51^f^±0.54
**HF-30**	10.63^ab^±0.40	2.95^c^±0.12	19.53^c^±0.05	3.23^c^±0.13	14.48^c^±0.15	2.54^ab^±0.01	17.02^c^±0.16	57.27^g^±0.26	382.11f^g^±0.28
**HF-35**	9.76^c^±0.08	3.33^b^±0.01	20.24^b^±0.10	3.66^ab^±0.24	16.13^b^±0.03	2.70^a^±0.18	18.83^b^±0.22	53.94^h^±0.34	380.64^g^±0.25
**HF-40**	9.73^c^±0.28	3.57^a^±0.03	20.87^a^±0.03	3.98^a^±0.16	18.71^a^±0.11	2.78^a^±0.05	21.49^a^±0.17	50.09^i^±0.49	376.92^h^±0.45
**HC-2.5**	10.74^ab^±0.01	0.97^ij^±0.02	15.21^i^±0.02	1.76^h^±0.07	3.70^ij^±0.16	1.59^e^±0.04	5.29^kl^±0.21	76.77^ab^±0.52	398.22^a^±0.02
**HC-5**	10.14^abc^±0.01	1.14^hi^±0.04	15.39^hi^±0.08	2.27^f^±0.03	4.71^hi^±0.16	1.67^e^±0.15	6.38^ji^±0.31	74.82^b^±0.48	398.59^a^±0.52
**HC-7.5**	10.87^a^±0.08	1.27^gh^±0.01	15.63^h^±0.05	2.44^ef^±0.05	5.65^gh^±0.16	1.74^de^±0.05	7.39^hi^±0.21	73.27^bc^±0.26	397.42^ab^±0.73
**HC-10**	10.47^abc^±0.10	1.47^fg^±0.04	16.14^g^±0.26	2.61^e^±0.12	6.38^g^±0.15	1.83^de^±0.01	8.21^gh^±0.16	71.57^c^±0.33	396.63^bc^±0.44

*carbohydrate content calculated by difference. d.m.–dry mass. w.m.–wet mass. CON–control sample. HF–hemp flour. HC–hemp cake. nd–not detected. IDF–insoluble dietary fiber. SDF–soluble dietary fiber. TDF–total dietary fiber. Means (n = 3) with different letters in the same column are significantly different (P < 0.05).

The protein content in samples with hemp flour increased by 6% for HF-5 to 43% for HF-40 compared to the CON sample. For the addition of hemp cake the increase is between 4% for sample HC-2.5 and 10% for sample HC-10 compared to the CON sample. The definition of the European Union says that a high-protein product is a product where at least 20% of energy comes from protein [28]. This criterion was met by samples HF-30, HF-35, and HF-40 with their protein content of 19.53–20.87% d.m. and 20.44–22.15% energy from protein. A similar value was obtained by Pojić et al. [17]. These studies showed that the addition of hemp flour to wheat bread at the level of 20% gives product in which 19.76% of the energy provided by protein. The protein content in the samples with the same addition of the hemp flour and hemp cake, i.e. HF-5 and HC-5 and HF-10 and HC-10, did not differ statistically. The fat content varied between 1.64 and 3.98% d.m. in HF-5 and HF-40, respectively, in the samples of pasta with hemp flour and between 1.76–2.61 in HC-2.5 and HC-10, respectively in the hemp cake-supplemented pasta samples. The addition of hemp flour at the level of 40% resulted in more than tripling of the fat content in relation to the control sample, while the highest addition of hemp cake (HC-10) increased the content of this macronutrient by 40%. Pasta with hemp raw materials can be an important source of dietary fiber in the diet. Most countries recommend a daily fiber intake of 25–35 g for adults [29]. A portion (75 g) of pasta with 30% hemp flour can cover up to 50% of the daily dietary fiber requirement. The ratio of soluble fiber to insoluble fiber in food should be approx. 1:2 [30]. In the present study, this ratio ranged from 1.93:1 for the CON to 6.73:1 for the HF-40. Hemp pasta is therefore not a good source of soluble fiber, since the insoluble dietary fiber is the predominant fraction. Therefore, when composing meals from hemp pasta, an adequate supply of the soluble fiber fraction should be taken into account. The mineral content of the CON sample was 0.75% d.m. The addition of 30% hemp flour increases this content about 4 times while 10% hemp cake additive about 1.8 times. The energy value of the samples decreases with the addition of hemp raw materials, which is mainly due to the increased content of dietary fiber. This parameter for samples with hemp flour decreases from 1% for HF-5 to 5% for HF-40 compared to CON. A decrease in the energetic value of pasta with the addition of high-fiber raw material was also noted by Michalak-Majewska et al. [31] in pasta with onion skin.

An important aspect of the safety of hemp food consumption is the THC and CBD content in the products. The content of the cannabinoids in enriched pasta samples is presented in Table 4. Depending on the country, the permitted intake of THC with food varies considerably. According to the data provided by Fresh Hemp Foods Ltd. [11], the daily intake of THC with food for a person with an average body weight of 70 kg should be in the range of 70–490 μg. Taking into account that the daily intake of pasta converted into dry product should be 75 g [32], the intake of the recommended portion prepared in the trials is associated with a supply of THC ranging from 27 μg for sample HF-5 to 424 μg for sample HC-10. The CBD content in all trials also met the set standards of less than 20 mg per day for an adult [14], which indicates the health safety of the products.

**Table 4:** The content of Δ-9-tetrahydrocannabinol (THC) and cannabidiol (CBD) in pasta samples.

Pasta sample	THC	CBD
	μg/100 g w.m.	
**CON**	nd	nd
**HF-5**	36.00^l^±1.00	53.00^l^±2.00
**HF-10**	72.00^k^±3.00	107.00^j^±3.00
**HF-15**	100.00^j^±3.00	160.00^h^±2.00
**HF-20**	144.00^h^±0.50	214.00^f^±4.00
**HF-25**	180.00^g^±2.00	267.00^d^±5.00
**HF-30**	221.00^f^±4.00	321.00^c^±6.00
**HF-35**	255.00^e^±5.00	374.00^b^±2.00
**HF-40**	288.00^c^±2.00	428.00^a^±3.00
**HC-2.5**	141.00^i^±3.00	56.00^k^±3.00
**HC-5**	282.00^d^±3.00	111.00^i^±4.00
**HC-7.5**	423.00^b^±2.00	167.00^g^±5.00
**HC-10**	565.00^a^±1.00	227.00^e^±1.00

CON–control sample. HF–hemp flour. HC–hemp cake. nd–not detected. THC - Δ-9-tetrahydrocannabinol. CBD–cannabidiol. Means (n = 3) with different letters in the same column are significantly different (P < 0.05).

The minimal cooking time increased with the addition of the hemp cake and the hemp flour. A similar trend was noted by Sykut-Domańska et al. [27], who enriched pasta with coconut residue and flour, and Zarzycki et al. [26] who fortified pasta with flaxseed flour and cake. This trend may be a result of an increase in the TDF content in the sample. Dietary fiber, showing high water absorption and competing for water with starch, impedes swelling and pasting of starch granules. As a result, the cooking time of the pasta is prolonged.

The weight increase index of the tested samples exceeded 2.0 (Table 5). In the present study, this index increased as the proportion of hemp flour increased up to 20% and then decreased. It can be concluded that a high proportion of hemp flour is accompanied by a significant weakening of the gluten network. This may be caused by the weakening of the gluten matrix as a result of the addition of hemp raw materials, which have high dietary fiber and protein content, but do not contain gluten. It should be noted that the main components of hemp seed proteins—soluble in water albumins and soluble in salt solution globulins (edestin), weaken the protein matrix and affected the deterioration of pasta quality. A similar trend was observed by Teterycz et al. [23] in pasta with the addition of legume seeds flour. The same argument may explain higher cooking losses. The losses in all the samples did not exceed the required 8%, but the value of this parameter increased significantly with the addition of hemp raw materials, with greater differences recorded for samples supplemented with the hemp cake. A loss of 5.45% of d.m. was recorded for the sample with 20% of the hemp flour and only 7.5% of the hemp cake. The higher values of cooking losses may be associated with the larger particle sizes of the hemp cake, compared to the hemp flour. Incorporated to the pasta high-fiber raw materials, characterized by high particle size may inhibit the formation of the gluten matrix in the pasta dough. During cooking, water penetrates the pasta structure more easily and the uncovered starch is more susceptible to leaching, thus increasing cooking losses [19]. A similar tendency was noted by Sobota et al. [33] in pasta enriched with vegetable components. The authors noted higher cooking losses in the case of pasta enriched with vegetable powders, which were characterized by higher dietary fiber content and higher granulation than vegetable concentrates. Sykut-Domańska et al. [27], who enriched pasta with coconut residue after oil extraction, and Sobota et al. [19], who supplemented pasta with wheat bran, reported a similar trend.

**Table 5:** Cooking quality of pasta samples.

Pasta sample	Minimal cooking time	Weight increase index	Cooking loss
	min		% d.m.
**CON**	6.00^f^±0.00	2.11^cd^±0.03	4.74^g^±0.10
**HF-5**	6.50^e^±0.00	2.11^cd^±0.08	5.01^efg^±0.10
**HF-10**	7.00^d^±0.25	2.17^abc^±0.02	5.17^def^±0.03
**HF-15**	7.50^c^±0.25	2.24^ab^±0.01	5.33^de^±0.10
**HF-20**	8.00^b^±0.25	2.25^a^±0.01	5.45^cd^±0.07
**HF-25**	8.50^a^±0.25	2.21^abc^±0.07	5.77^bc^±0.11
**HF-30**	8.50^a^±0.00	2.12^bcd^±0.05	5.91^ab^±0.16
**HF-35**	8.50^a^±0.25	2.09^cd^±0.13	6.05^ab^±0.10
**HF-40**	8.50^a^±0.00	2.04^d^±0.12	6.26^a^±0.05
**HC-2.5**	6.50^e^±0.25	2.21^abc^±0.07	4.89^fg^±0.20
**HC-5**	7.50^c^±0.25	2.24^a^±0.07	5.17^def^±0.25
**HC-7.5**	7.50^c^±0.00	2.24^a^±0.06	5.45^cd^±0.12
**HC-10**	8.00^b^±0.25	2.26^a^±0.11	5.51^cd^±0.27

CON–control sample. HF–hemp flour. HC–hemp cake. Means (n = 3) with different letters in the same column are significantly different (P < 0.05).

The value of WSI for pasta samples enriched with more than 5% of hemp flour was higher, compared to the control sample (Table 6). In the case of HC, no effect of the additive on the WSI value was noted. However, the addition of hemp flour as well as hemp cake significantly affected the water absorption index (WAI) of the samples. The WAI value in the hemp flour-supplemented samples increased up to the 20% addition of HF and then decreased. A similar tendency was noted for the weight increase index (Table 5). This may be related to the weakening of the protein network of the pasta, which then cannot absorb more water. In the case of hemp cake-supplemented pasta WAI increased slightly when the share of the hemp component increased from 2.5 to 10%. The WSI results did not differ significantly for the same HF and HC additives (5%, 10%).

**Table 6:** Physical properties of raw materials and pasta samples.

Samples	WSI	WAI
	% d.m.	%
**Semolina**	4.62^b^±0.08	79.61^b^±0.61
**Hemp flour**	9.37^a^±0.03	133.55^a^±1.83
**Hemp cake**	9.11^a^±0.09	128.89^a^±0.49
**CON**	6.94^e^±0.04	131.84^f^±3.93
**HF-5**	7.14^de^±0.08	173.21^de^±4.59
**HF-10**	7.63^abc^±0.08	187.02^cd^±2.53
**HF-15**	7.86^a^±0.12	208.98^b^±0.55
**HF-20**	8.00^a^±0.14	235.09^a^±2.30
**HF-25**	8.05^a^±0.01	220.57^ab^±3.82
**HF-30**	7.81^ab^±0.04	205.34^bc^±2.30
**HF-35**	7.71^ab^±0.19	168.27^de^±3.40
**HF-40**	7.66^ab^±0.30	158.03^e^±2.85
**HC-2.5**	7.07^e^±0.11	170.07^de^±3.09
**HC-5**	7.18^cde^±0.05	176.61^de^±3.43
**HC-7.5**	7.37^bcde^±0.15	182.18^d^±2.13
**HC-10**	7.61^bcde^±0.05	184.87^d^±1.43

CON–control sample. HF–hemp flour. HC–hemp cake. Means (n = 3) with different letters in the same column are significantly different (P < 0.05).

The texture parameters of cooked pasta obtained in TPA are presented in Table 7. The results indicate that neither the raw materials used in the model nor their quantity affected the springiness values of the products in relation to the CON sample. All pasta samples were very springy. This was also noticed by the consumer panel, who also evaluated this parameter very highly in all samples (Table 8). The hardness of the pasta ranged from 2.93 N for CON to 5.80 N for HC-10. This parameter increased in the hemp flour addition range from 5 to 25% and then decreased in the range of 30–40%. A similar trend was also observed in the case of pasta chewiness, WAI value, and weight increase index. In accordance with Monteiro et al., [34] pasta samples with higher protein content become harder. However, our research noted that this happens only to a certain level of addition. By introducing hemp flour into pasta, we increase not only the protein content but also the fiber content, which can damage the structure of pasta and thus lower its texture parameters [35]. The samples with the hemp cake were characterized by significantly higher hardness and chewiness than the pasta with the same content of hemp flour. Due to its higher granulation, it caused the denser twisting of the fusilli form, which is clearly visible in the (S1 and S2 Figs). This, in turn, resulted in the higher hardness of the product. Cohesiveness indicates coherence of the material. In the case of the pasta with the hemp flour, the cohesiveness value decreased with the addition of this material. This is related to higher water absorption and higher fiber content. The cohesion of the pasta with the hemp cake was lower, compared to samples with the same percentage of the hemp flour. The larger particles of this raw material disturbed the structure of the pasta and reduced its cohesiveness.

**Table 7:** Texture parameters of cooked pasta samples.

Pasta sample	Hardness	Springiness	Chewiness	Cohesiveness
	N		N	
CON	2.93^e^±0.23	0.991^a^±0.006	1.98^de^±0.15	0.66^a^±0.01
HF-5	2.98^e^±0.21	0.991^a^±0.005	1.93^de^±0.15	0.66^a^±0.01
HF-10	3.70^cde^±0.23	0.991^a^±0.006	2.35^bcde^±0.1 5	0.63^ab^±0.01
HF-15	4.00^cde^±0.23	0.991^a^±0.006	2.38^bcde^±0.15	0.61^abc^±0.01
HF-20	4.32^bcd^±0.21	0.992^a^±0.005	2.49^bcd^±0.15	0.58^bcd^±0.01
HF-25	4.34^bcd^±0.24	0.991^a^±0.006	2.49^bcd^±0.17	0.58^bcd^±0.01
HF-30	3.69^cde^±0.23	0.987^a^±0.006	2.06^cde^±0.16	0.58^bcd^±0.01
HF-35	3.41^de^±0.24	0.989^a^±0.006	1.88^de^±0.17	0.56^cd^±0.01
HF-40	2.94^e^±0.23	0.987^a^±0.006	1.68^e^±0.16	0.56^cd^±0.01
HC-2.5	4.86^abc^±0.27	0.990^a^±0.007	2.92^ab^±0.18	0.60^abc^±0.02
HC-5	5.42^ab^±0.27	0.991^a^±0.007	2.88^abc^±0.18	0.53^d^±0.02
HC-7.5	5.61^a^±0.28	0.991^a^±0.007	3.03^ab^±0.18	0.52^d^±0.02
HC-10	5.80^a^±0.24	0.992^a^±0.006	3.38^a^±0.17	0.52^d^±0.01

CON–control sample. HF–hemp flour. HC–hemp cake. Means (n = 5) with different letters in the same column are significantly different (P < 0.05).

**Table 8:** Sensory analysis of uncooked and cooked pasta samples.

Pasta sample	Appearance	Color	Smell	Average score	Appearance	Color	Taste	Smell	Hardness	Adhesiveness	Springiness	Average score
**CON**	5.00^a^±0.00	4.89^a^±0.33	5.00^a^±0.00	4.96^a^±0.11	4.78^a^±0.44	4.89^a^±0.33	4.89^a^±0.33	4.89^a^±0.33	4.89^a^±0.33	4.67^a^±0.5	4.78^a^±0.44	4.83^a^±0.27
**HF-5**	4.78^ab^±0.67	4.67^a^±0.50	4.56^ab^±0.53	4.67^ab^±0.33	4.78^a^±0.44	4.78^ab^±0.44	4.33^ab^±0.50	4.44^ab^±0.53	4.89^a^±0.33	4.89^a^±0.33	4.78^a^±0.44	4.70^ab^±0.26
**HF-10**	4.44^abc^±0.53	4.33^a^±0.50	4.78^ab^±0.44	4.52^abc^±0.34	4.78^a^±0.44	4.56^abc^±0.53	4.33^ab^± 0.71	4.22^abc^±0.67	4.89^a^±0.33	4.89^a^±0.33	4.78^a^±0.44	4.63^ab^±0.28
**HF-15**	4.56^ab^±0.53	4.44^a^±0.53	4.44^ab^±0.53	4.48^abc^±0.41	4.56^a^±0.53	4.33^abc^±0.50	4.11^abc^± 0.33	4.33^abc^±0.71	4.78^a^±0.44	4.89^a^±0.33	4.89^a^±0.33	4.56^abc^±0.24
**HF-20**	4.56^ab^±0.53	4.67^a^±0.50	4.22^ab^±0.67	4.48^abc^±0.47	4.44^ab^±0.53	4.44^abc^±0.73	3.78^bcd^± 0.78	3.89^abc^±0.60	4.67^a^±0.50	4.78^a^±0.44	4.78^a^±0.44	4.30^bcd^±0.24
**HF-25**	4.56^ab^±0.53	4.67^a^±0.50	4.22^ab^±0.67	4.48^abc^±0.47	4.33^ab^±0.50	4.11^abc^±0.60	3.78^bcd^± 0.73	3.89^abc^±0.60	4.78^a^±0.44	4.89^a^±0.33	4.89^a^±0.33	4.33^bcd^±0.23
**HF-30**	4.22^abc^±0.67	4.44^a^±0.73	4.33^ab^±0.71	4.33^abc^±0.29	4.22^ab^±0.67	3.89^bc^±0.60	3.44^bcd^±0.50	3.56^bc^±0.73	4.78^a^±0.44	4.89^a^±0.33	4.78^a^±0.44	4.16^cd^±0.15
**HF-35**	4.00^bc^±0.71	4.11^a^±0.60	4.00^b^±0.50	4.04^bc^±0.42	3.89^ab^±0.6	3.67^c^±0.87	3.44^bcd^± 0.60	3.56^bc^±0.73	4.89^a^±0.33	4.89^a^±0.33	4.89^a^±0.33	4.13^cd^±0.38
**HF-40**	3.56^c^±0.73	4.22^a^±0.67	4.00^b^±0.50	3.93^c^±0.32	3.56^b^±0.88	3.78^c^±0.67	3.11^cd^± 0.53	3.33^c^±0.87	4.89^a^±0.33	4.78^a^±0.44	4.89^a^±0.33	4.10^d^±0.33
**HC-2.5**	4.33^abc^±0.71	4.11^a^±0.60	4.44^ab^±0.73	4.30^abc^±0.54	4.44^ab^±0.53	4.22^abc^±0.67	4.00^abcd^± 0.71	4.44^ab^±0.53	4.78^a^±0.44	4.67^a^±0.50	5.00^a^±0.00	4.51^abcd^±0.20
**HC-5**	4.22^abc^±0.83	4.22^a^±0.83	4.33^ab^±0.71	4.26^bc^±0.64	4.22^ab^±0.67	4.33^abc^±0.71	3.78^bcd^± 0.83	4.11^abc^±0.78	4.89^a^±0.33	4.78^a^±0.44	4.89^a^±0.33	4.43^abcd^±0.34
**HC-7.5**	4.11^abc^±0.78	4.33^a^±0.50	4.22^ab^±0.83	4.22^bc^±0.62	4.22^ab^±0.67	4.11^abc^±0.78	3.78^bcd^± 0.67	4.00^abc^±0.71	4.89^a^±0.33	4.78^a^±0.44	5.00^a^±0.00	4.40^abcd^±0.30
**HC-10**	4.11^abc^±0.33	4.00^a^±0.50	4.22^ab^±0.67	4.11^bc^±0.37	4.11^ab^±0.60	3.78^c^±0.44	3.78^bcd^± 0.83	4.00^abc^±0.50	4.78^a^±0.44	4.78^a^±0.44	5.00^a^±0.00	4.32^bcd^±0.21

CON–control sample. HF–hemp flour. HC–hemp cake. Means (n = 20) with different letters in the same column are significantly different (P < 0.05).

Consumer acceptance is one of the most important criteria for evaluating a newly developed food product. The consumer panel selected for the survey assessed the samples at a satisfactory level (Table 8). The lowest scores for taste, which is the most important food feature according to consumers [36], were recorded for the HF-40 sample, but none of the taste scores were lower than 3 which may indicate satisfactory consumer acceptance. During the evaluation of this parameter, the panelists reported a bitter taste of the product. It may be associated with the presence of saponins in cannabis material [37]. The average consumer assessment of uncooked pasta indicates that only samples HF-40 received a score below 4, and for cooked pasta, all the notes were above 4, which is a very promising result.

## Conclusion

The results of the research indicate the possibility of using hemp components to improve the nutritional value of pasta while maintaining its safety. It should be concluded, however, that the addition of hemp flour (HF) rather than hemp cake (HC) is a better solution because the former component facilitates the technological process and thus increases the possibility of fortification. Both hemp raw materials allow enriching pasta with protein, dietary fiber, mainly its insoluble fraction and minerals. Taking into account the chemical composition and quality parameters of pasta, the optimal addition to pasta is 30% for hemp flour (HF-30) and 10% for hemp cake (HC-10). Pasta with 30% addition of hemp flour can be described as a high-protein and high-fiber product and at the same time characterized by satisfactory organoleptic properties and good cooking qualities. THC and CBD content in all enriched pasta was on a safe level. Although pasta with hemp raw materials contains more protein than conventional pasta, it seems advisable to conduct further research to determine the digestibility of protein and bioactive peptides content in obtained products.

## Supporting information

S1 Fig The pasta samples enriched with hemp flour. (DOCX)

S2 Fig The pasta samples enriched with hemp cake. (DOCX)
