# Supplementary figures and images for: Hemp seed (Cannabis sativa L.) enriched pasta: Physicochemical properties and quality evaluation
Source: PLoS One. 2021 Mar 18;16(3):e0248790. doi: 10.1371/journal.pone.0248790 (PMC7971538; doi:10.1371/journal.pone.0248790)

| **CON** | **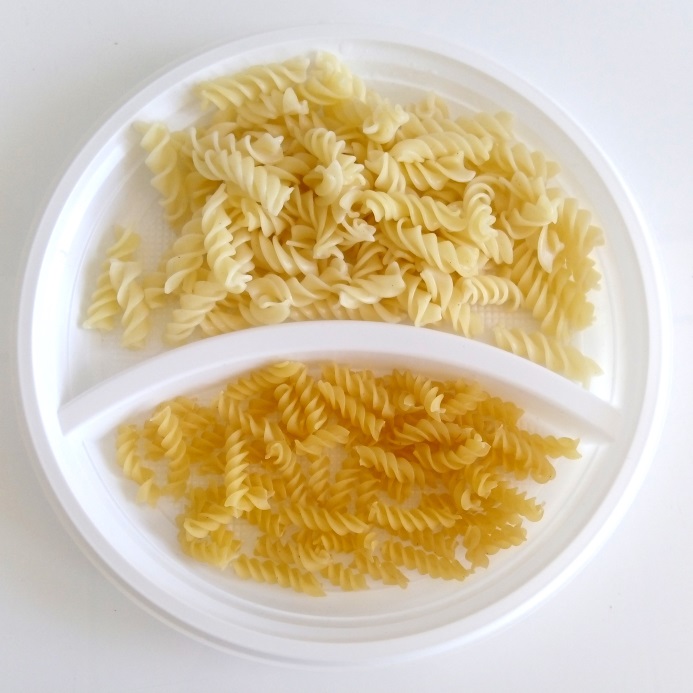** | **HF-25** | **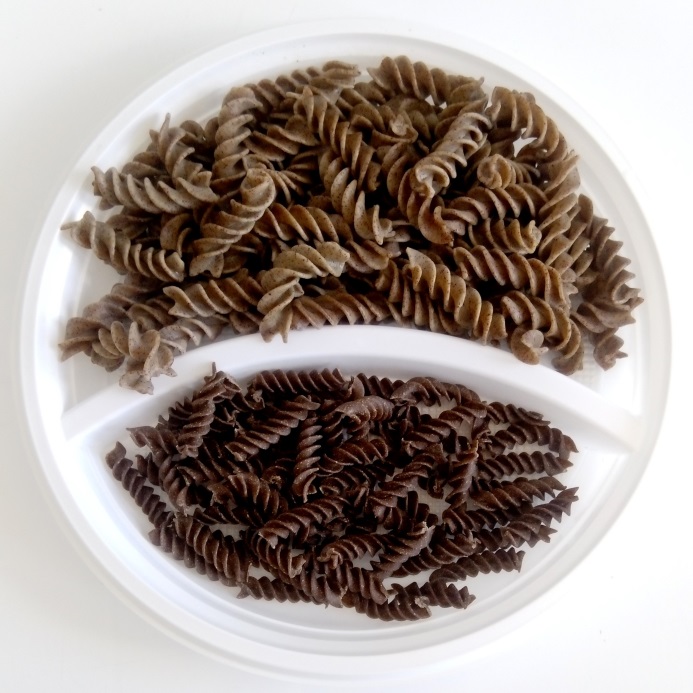** |
| --- | --- | --- | --- |
| **HF-5** | **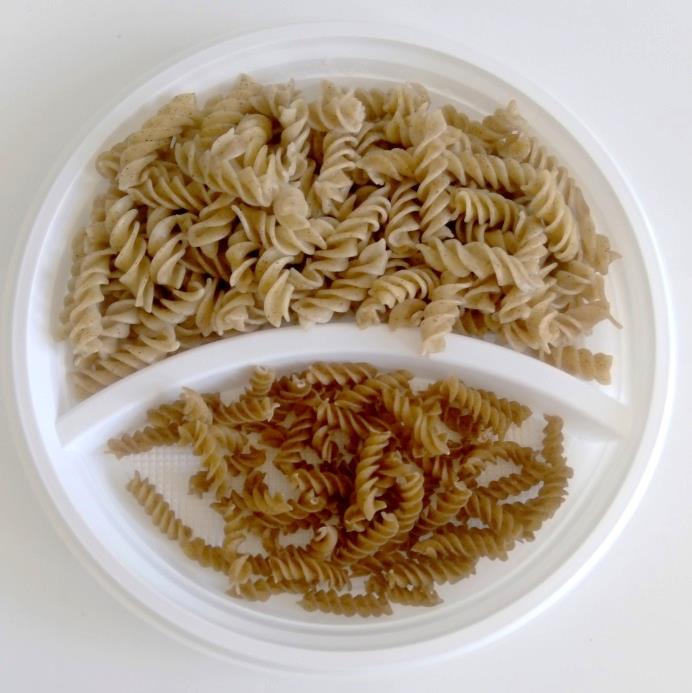** | **HF-30** | **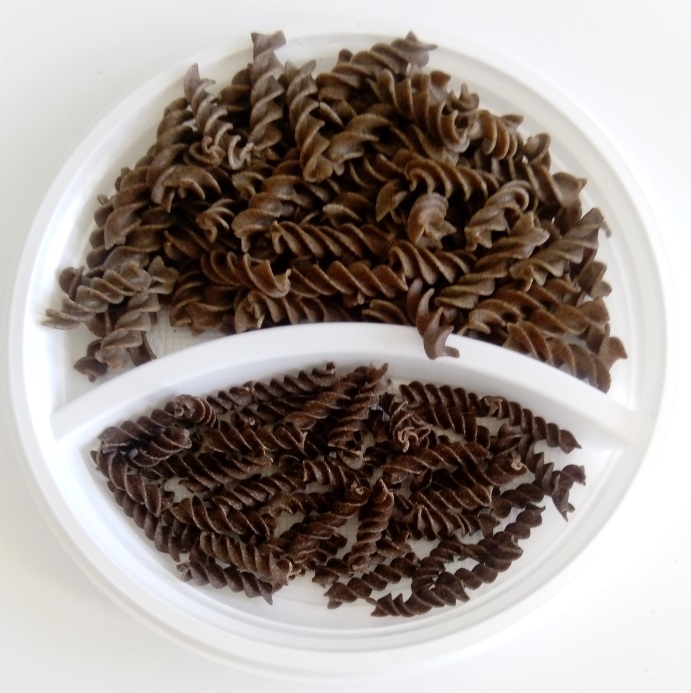** |
| **HF-10** | **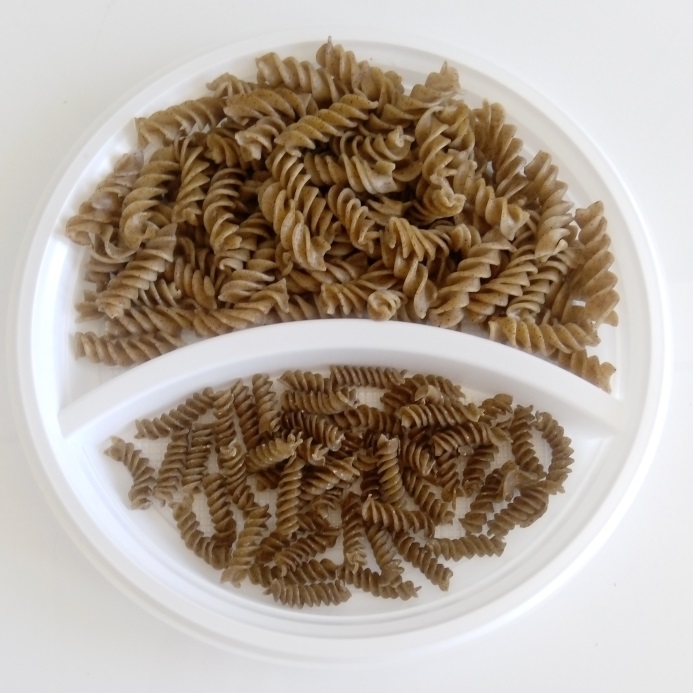** | **HF-35** | **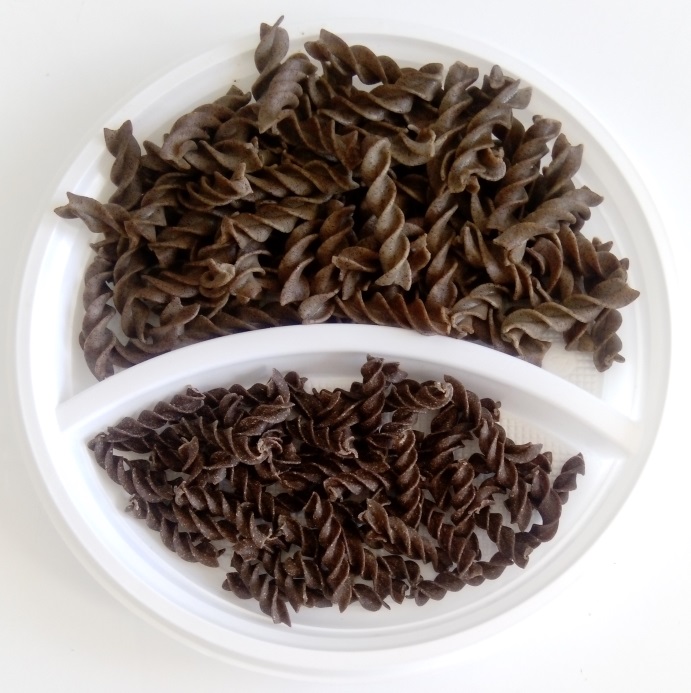** |
| **HF-15** | **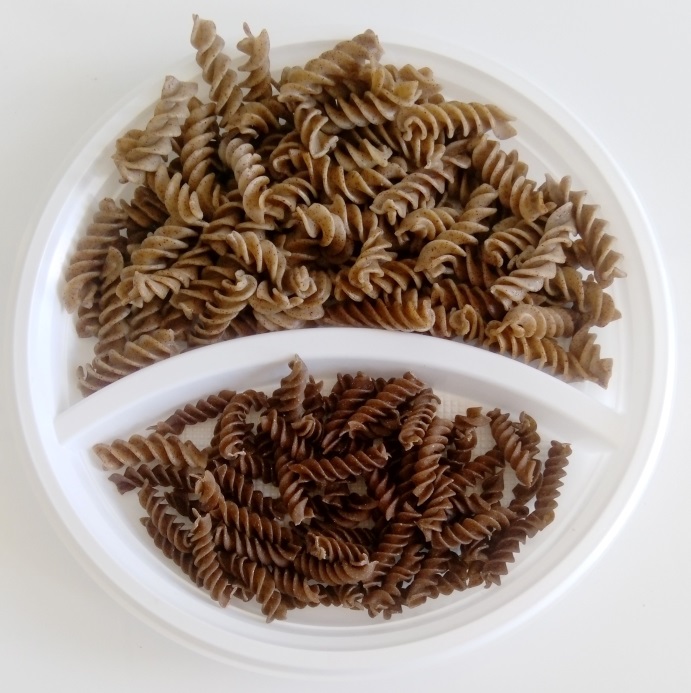** | **HF-40** | **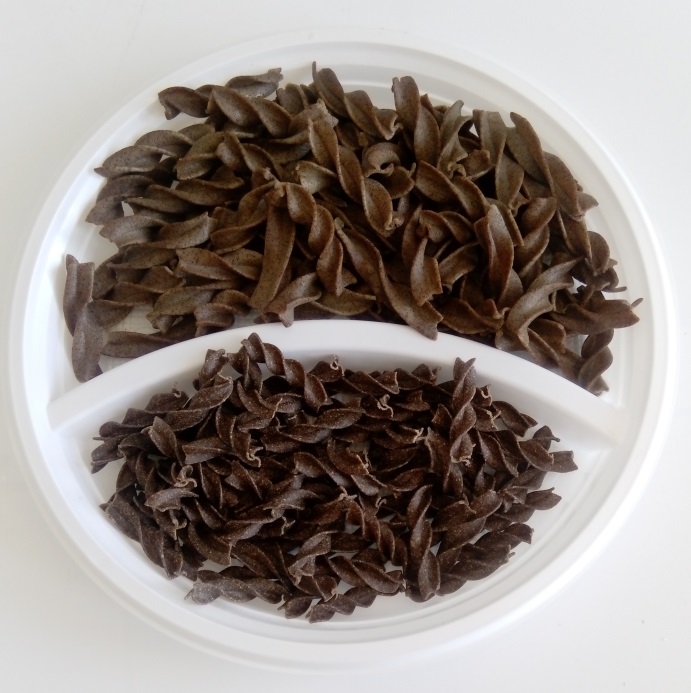** |
| **HF-20** | **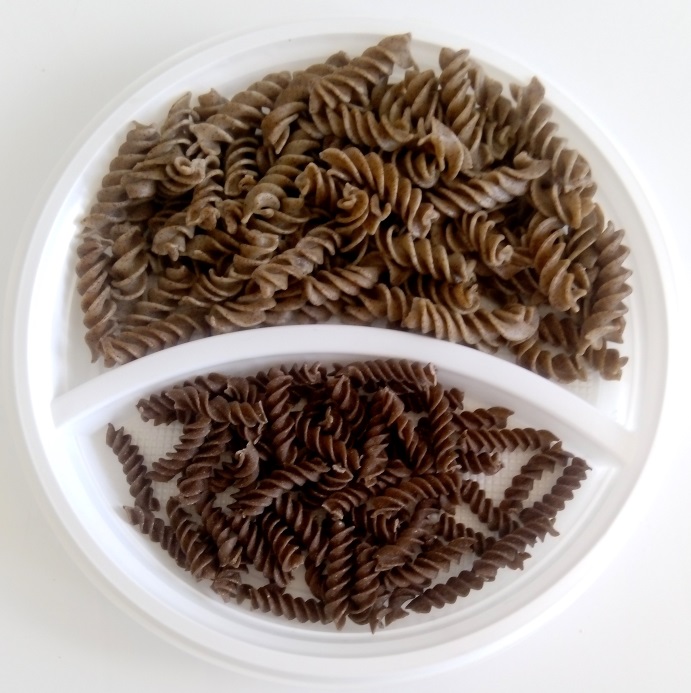** |  |  |

S1 Fig. The pasta samples enriched with hemp flour

Supplement: S1 Fig — (DOCX) [file pone.0248790.s001.docx]

| **CON** | **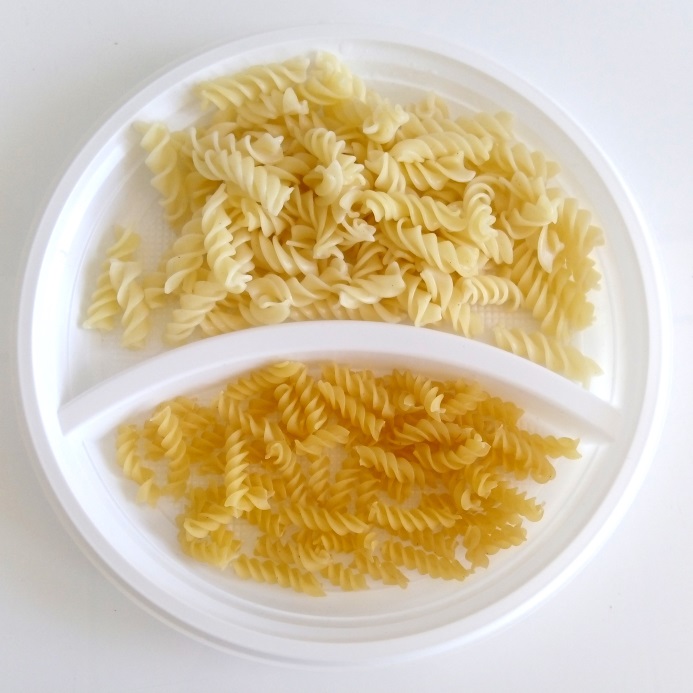** |
| --- | --- |
| **HC-2.5** | **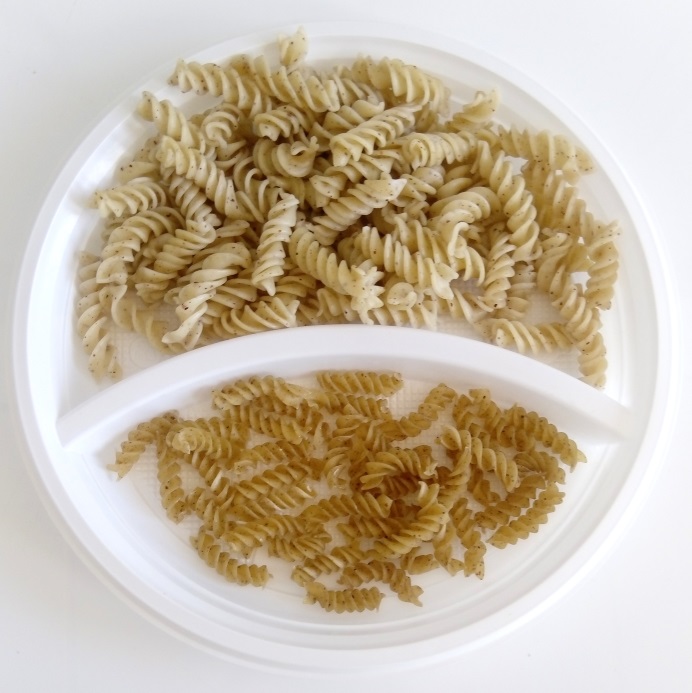** |
| **HC-5** | **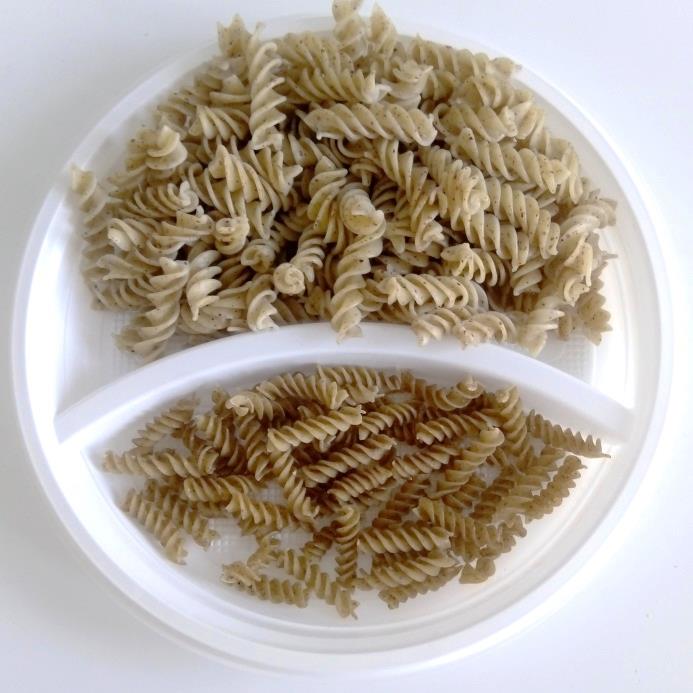** |
| **HC-7.5** | **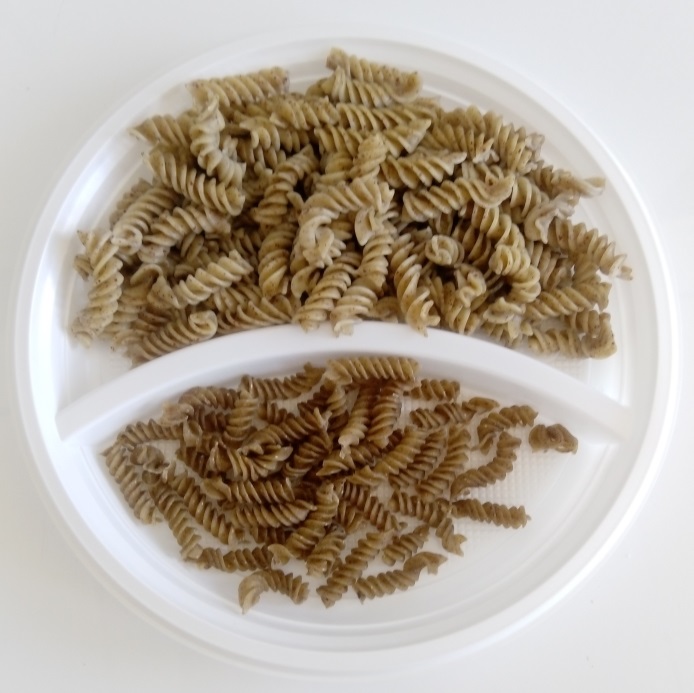** |
| **HC-10** | **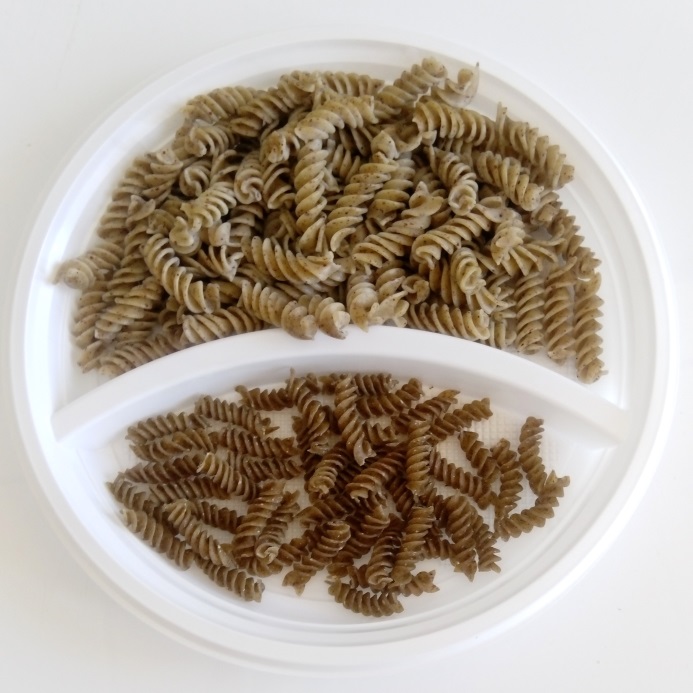** |

S2 Fig. The pasta samples enriched with hemp cake.

Supplement: S2 Fig — (DOCX) [file pone.0248790.s002.docx]
